# Insights into ALS‐inhibiting herbicide resistance in *Poa annua* in an arable cropping system

**DOI:** 10.1002/ps.70121

**Published:** 2025-08-12

**Authors:** Alwarnaidu Vijayarajan Vijaya Bhaskar, Iniya Vijayakumar, Joel Torra, Fabian Runge, Michael Hennessy, Patrick Dermot Forristal

**Affiliations:** ^1^ Teagasc Oak Park Research Centre Carlow Ireland; ^2^ South East Technological University, Carlow Campus Carlow Ireland; ^3^ Department of Agricultural and Forest Sciences and Engineering University of Lleida—Agrotecnio CERCA Centre Lleida Spain; ^4^ IDENTXX GmbH Stuttgart Germany

**Keywords:** Acetolactate synthase inhibitors, annual bluegrass, target‐site mutations, winter wheat

## Abstract

**BACKGROUND:**

Dependence on a single herbicide type to control a broad‐spectrum of weeds has resulted in cases of resistance to acetolactate synthase (ALS) inhibitors in weeds normally considered lower priority such as *Poa annua*, being recorded in winter wheat fields in Ireland. This study characterizes resistance to ALS‐inhibiting sulfonylureas (SU), sulfonylamino‐carbonyl‐triazolinones (SCT) and triazolopyrimidine (TP) herbicides in five resistance‐suspect populations (POAAN‐R1 to POAAN‐R5).

**RESULTS:**

Variability in cross‐resistance levels were noted in the POAAN‐R populations, correlating with target‐site mutations and specific amino acid substitutions. Mutations Pro‐197‐Thr in POAAN‐R1, Pro‐197‐Thr + Trp‐574‐Leu in POAAN‐R4, and Trp‐574‐Leu in POAAN‐R5 confer high levels of resistance to SU, SU + SCT and TP herbicides (GR_50_ resistance index, RI >10). In POAAN‐R2, Pro‐197‐Gln confers high resistance to SU and SU + SCT (RI >10) and low resistance to TP (RI = 3.2). POAAN‐R3 with Pro‐197‐Thr + Pro‐197‐Gln, confers moderate resistance to SU and SU + SCT (RI >5) and high resistance to TP (RI >10). All sequenced plants had homozygous mutations that evolved independently in *ALS*1 (Pro‐197‐Gln) or *ALS*2 (Pro‐197‐Thr or Trp‐574‐Leu) isoforms. Among the alternative non‐residual herbicides evaluated, clethodim and glyphosate effectively controlled *P. annua* populations.

**CONCLUSION:**

Pro‐197‐Thr and/or Pro‐197‐Gln, as well as the combination of Pro‐197‐Thr + Trp‐574‐Leu were identified for the first time in *P. annua* in this study. As cultural/non‐chemical management of *P. annua* is challenging, the primary control in cereal crops will likely be the use of non‐ALS herbicide modes of action, including the application of pre‐emergence or autumn residual‐type herbicides. © 2025 The Author(s). *Pest Management Science* published by John Wiley & Sons Ltd on behalf of Society of Chemical Industry.

## Graphical abstract

Variable cross‐resistance to ALS inhibitors in five *Poa annua* populations from wheat fields in Ireland caused by different TSR mutations.

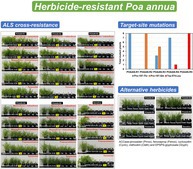

## 1 INTRODUCTION

In Ireland, *Poa annua* L. (annual bluegrass or meadow grass) is widespread in arable crop fields and field boundaries, facilitated by the mild wet Atlantic climate and a continuous cropping system that has replaced a previous mixed farming (grazing livestock and annual crops in rotation) base.^1^, ^2^, ^3^ It is primarily an annual, self‐pollinating allotetraploid (2*n* = 4*x* = 28) weed, with high fecundity and seed viability that forms a persistent seed bank.^4^, ^5^ Despite its widespread distribution in crop fields, *P. annua* has historically been easy to control using a variety of herbicide options, and consequently was ranked as a low priority cereal grass weed in Ireland.^1^ In the UK, which has a climate broadly similar to Ireland, the economic threshold for *P. annua* in winter cereals is 50 plants m^−2^,^6^ which highlights that its yield impact is not as serious as that of other, higher priority, grass weeds in this region.^1^

Spring barley (120 000 ha), winter wheat (60 000 ha) and winter barley (60 000 ha) are the dominant cereal crops in Ireland.^7^ Herbicides are central to cereal weed control strategies, especially in winter wheat.^8^ Wetter autumns, with monthly rainfall between 78.6 to 126.2 mm,^9^ combined with fewer windows for pre‐emergence spraying, and the ban on isoproturon in the EU in 2017 have led to increased use of acetolactate synthase (ALS)‐inhibiting herbicides applied post‐emergence in spring.^2^, ^3^ A number of ALS‐inhibiting herbicides are used on winter wheat, primarily to control the competitive grass weed *Bromus sterilis* L. (barren or sterile brome), such as sulfonylureas (SU, e.g., mesosulfuron or iodosulfuron), triazolopyrimidines (TP, e.g., pyroxsulam) and sulfonylamino‐carbonyl‐triazolinones (SCT, e.g., propoxycarbazone or thiencarbazone). These broad‐spectrum ALS inhibitors are commercially available only as co‐formulated herbicides, with SU (mesosulfuron + iodosulfuron) and SU + SCT (mesosulfuron + propoxycarbazone) registered in Ireland for controlling *P. annua* in winter wheat.^2^, ^3^ The repeated use of these ALS inhibitors as the sole method of weed control in winter wheat has increased, which poses resistance risks in both high priority but also lower priority weed species, including *P. annua*.^1^, ^2^, ^3^, ^10^

*Poa annua* ranks as the most common herbicide‐resistant weed globally, having developed resistance to 12 different modes of action, primarily in non‐arable crop settings (e.g., golf courses and turf).^11^ Target‐site resistance (TSR) mechanisms such as gene mutation and amplification, or non‐target‐site resistance (NTSR) mechanisms such as enhanced metabolism, reduced absorption and/or translocation of herbicides, have been identified in *P. annua*.^12^, ^13^, ^14^, ^15^, ^16^, ^17^, ^18^, ^19^, ^20^, ^21^, ^22^ Most ALS‐resistant *P. annua* cases have been associated with TSR mutations, typically Pro‐197, Trp‐574 or Ala‐205;^12^, ^15^, ^16^, ^20^ however, NTSR involvement has only been confirmed in one case where it occurred alongside TSR.^17^ In arable crops, only two cases of ALS‐resistant *P*. annua have been reported internationally (France in 2015 and Ireland in 2021).^2^, ^11^ Recently, some populations of Danish *P. annua*, mainly in continuous maize cropping, have been found to be resistant to ALS inhibitors.^23^ It is of note that *P. annua* exhibits natural resistance to some acetyl‐CoA carboxylase (ACCase) inhibitors, due to the inherited mutation at Ile‐1781 position on the ACCase enzyme.^2^, ^12^, ^24^ Despite this trait, clethodim (registered in Ireland for use in winter oilseed rape or beet) and haloxyfop still offer effective control of *P. annua*, due to polyploidy and gene dilution.^2^, ^25^, ^26^

In the previously confirmed case of ALS‐resistant *P. annua* in Ireland, the Trp‐574 mutation was found to cause cross‐resistance to SU (mesosulfuron + iodosulfuron) and TP (pyroxsulam, which is not registered for *P. annua* control in Ireland) herbicides, and no evidence of enhanced metabolism (NTSR) was found.^2^ Initially, this was considered an isolated case, but reports from the 2023 and 2024 Teagasc weed monitoring program (the Irish state‐funded research and farm advisory organization) suggest that resistance in *P. annua* may be more widespread requiring a more detailed understanding of the development of resistance. Preliminary screenings showed that 12 out of 14 suspected *P. annua* populations were not controlled by the standard SU application rate. Five resistant *P. annua* populations (POAAN‐R1 to POAAN‐R5) were selected for this study. The objectives of this study were to: (i) identify TSR mutations in POAAN‐R populations and pinpoint their location in gene variants, (ii) quantify the resistance and cross‐resistance levels to co‐formulated SU (mesosulfuron + iodosulfuron), SU + SCT (mesosulfuron + propoxycarbazone or mesosulfuron + thiencarbazone), and TP (pyroxsulam), as well as stand‐alone SCT (propoxycarbazone) ALS inhibitors, and (iii) assess the efficacy of alternative non‐ALS post‐emergence herbicides for controlling ALS‐resistant populations.

## 2 MATERIALS AND METHODS

### 2.1 Plant materials

Five resistant *P. annua* populations from different counties of Ireland were used in this study (Table 1). POAAN‐R1, POAAN‐R2, POAAN‐R3 and POAAN‐R5 survived field application of SU (mesosulfuron + iodosulfuron) at rates of 7.5 + 2.5 g active ingredient (ai) ha^−1^ or 12 + 4 g ai ha^−1^ (i.e., 0.5 or 0.8 times the recommended label rate of product Pacifica Plus, Bayer CropScience Ltd., Cambridge, UK). While POAAN‐R4 survived field application of combined SU (7.5 g ha^−1^ mesosulfuron +2.5 g ha^−1^ iodosulfuron) and TP (14.2 g ha^−1^ pyroxsulam; 0.75 times the recommended label rate of product Broadway Star, Corteva Agrisciences, Cambridge, UK). Seed samples were harvested and submitted by growers from at least 20 random plants as instructed, although the seed quantity was limited. The source fields for these populations had a history of intensive SU use for grass weed control. A previously known ALS‐sensitive population of *P. annua* (from WeberSeeds Botany & Ethnobotany, Vaals, Netherlands) was used as a sensitive (S) reference.^2^

**Table 1:** Population origin of *P. annua* (POAAN‐R) used for resistance analysis

Population code	Field position	County	Country	Harvested crop	Year
POAAN‐R1	52°26′ N–6°51′ W	Wexford	Ireland	Winter wheat	2023
POAAN‐R2	54°37′ N–6°35′ W	Armagh	Northern Ireland, UK	Winter wheat	2023
POAAN‐R3	53°19′ N–6°98′ W	Kildare	Ireland	Winter wheat	2023
POAAN‐R4	53°95′ N–6°55′ W	Louth	Ireland	Winter wheat	2024
POAAN‐R5	51°90′ N–8°45′ W	Cork	Ireland	Winter wheat	2024

### 2.2 Growing conditions

The dose–response and alternative herbicide assays were carried out on R and S seedlings grown in glasshouse conditions during the winters of 2023/2024 and 2024/2025. The experiments in 2023/2024 were conducted in 110 × 110 × 110 mm pots filled with a soil mix consisting of 70% loam, 20% horticultural grit, 10% peat (medium), and 2 g L^−1^ of Osmocote Mini™ (National Agrochemical Distributors Ltd., County Dublin, Ireland). Due to the unavailability of the original growing media, a similar soil texture product ‘Progrow’ soil mix (Enrich Environmental Ltd., County Meath, Ireland) containing 58% sand, 22% silt and 20% clay, was used in 90 × 90 × 100 mm pots for the 2024/2025 experiments. All pots were watered as needed throughout the experiment. The plants were grown in a glasshouse with 18 °C/12 °C (day/night) temperature regime at a photoperiod of 16 h with artificial lighting supplementation to maintain a minimum light intensity of 250 μ mol quanta m^−2^ s^−1^.

### 2.3 Dose–response to ALS inhibitors

At the two‐to‐four leaf stage (BBCH 12–14), the R and S populations were treated with a range of doses of ALS inhibitors (Table 2). Seven rates used for R populations: 0.25*x*, 0.5*x*, 1*x*, 1.5*x*, 2*x*, 4*x* and 8*x*, where *x* is the recommended label rate, and for the S population; 0.0625x, 0.125×, 0.25*x*, 0.5*x*, 1*x*, 1.5*x* and 2*x* were used. The experiment with co‐formulated herbicide treatments included four replicates (three R populations alongside a sensitive population in 2023/2024, and two R populations alongside a sensitive population in 2024/2025) (Table 1). The growing of the sensitive reference population in both soil mixes allowed confirmation of a similar response to herbicide use in both media. The stand‐alone herbicide treatment used three replicates for both R (2023 and 2024 populations) and S, all tested in a single media (Progrow soil) in 2024/2025. Treatments were applied using a Generation III Research Track Sprayer (DeVries Manufacturing, Hollandale, MN, USA) with a Teejet 8002‐EVS flat fan nozzle, delivering an output of 200 L ha^−1^ at a pressure of 250 kPa and track speed of 1.2 ms^−1^. The applied herbicide was allowed to dry on the foliage before the pots were returned to the glasshouse where watering resumed after 24 h. At 30 days post‐treatment, the above‐ground plant material was harvested from each replicate and shoot fresh weight was recorded. Fresh biomass was expressed as the percentage of the mean fresh biomass of the non‐treated controls.

**Table 2:** Co‐formulated and stand‐alone ALS‐inhibiting sulfonylureas (SU), sulfonylamino‐carbonyl‐triazolinones (SCT) and triazolopyrimidines (TP) herbicides used for dose–response assays in R and S populations of *P. annua.* The recommended label rate for each herbicide is highlighted in bold

Type	Chemical families	Trade name^a^,^†^	Source	Active ingredient (ai)^b^	Dose rates used (g ai ha^−1^)
Co‐formulated	SU	Pacifica® Plus	Bayer CropScience Ltd, Cambridge, UK	Mesosulfuron + iodosulfuron	0.9 + 0.3, 1.9 + 0.6, 3.8 + 1.3, 7.5 + 2.5, **15 + 5**, 22.5 + 7.5, 30 + 10, 60 + 20, 120 + 40 and 0
	SU + SCT	Monolith®	Bayer CropScience Ltd, Cambridge, UK	Mesosulfuron + propoxycarbazone	0.9 + 1.4, 1.9 + 2.8, 3.7 + 5.6, 7.4 + 11.1, **14.9 + 22.3**, 22.3 + 33.4, 29.7 + 44.6, 59.4 + 89.1, 118.8 + 178.2 and 0
		Incelo®	Bayer CropScience Ltd, Cambridge, UK	Mesosulfuron + thiencarbazone	0.9 + 0.3, 1.9 + 0.6, 3.7 + 1.2, 7.4 + 2.6, **14.9 + 5.0**, 22.3 + 7.4, 29.7 + 9.9, 59.4 + 19.8, 118.8 + 39.6 and 0
	TP	Broadway® Star	Corteva Agrisciences, Cambridge, UK	Pyroxsulam	1.2, 2.4, 4.7, 9.4, **18.8**, 28.1, 37.5, 75.0, 150.1 and 0
Stand‐alone	SCT	Attribut®	Bayer CropScience Ltd, Monheim, Germany	Propoxycarbazone	4.4, 8.8, 17.5, 35, **70**, 105, 140, 280, 560 and 0

^†^ Products Incelo or Atrribut are not registered for use in Irish cereal crops.

^a^ The amidosulfuron component in Pacifica Plus, and the florasulam component in Broadway Star, are included primarily to provide broad‐leaved weed control.^2^ Therefore the efficacy of mesosulfuron + iodosulfuron in Pacifica Plus and pyroxsulam in Broadway Star on *P. annua* was evaluated.

^b^ Treatments with mesosulfuron + iodosulfuron, mesosulfuron + propoxycarbazone or mesosulfuron + thiencarbazone were applied with 1% v/v Biopower (alkylethersulfate sodium salt) adjuvant (Bayer CropScience Ltd, Cambridge, UK); treatments with pyroxsulam were applied with 1% v/v Kantor (alkoxylated triglycerides) adjuvant (Interagro Ltd., Hertfordshire, UK).

### 2.4 Genetic analyses

Leaf samples from *P. annua* plants were taken from the preliminary screening and air‐dried at room temperature. The DNA isolation, amplification and sequencing was carried out by IDENTXX GmbH (Stuttgart, Germany).^3^ Leaf segments (∼0.5 cm^2^) from eight plants of the R populations that survived mesosulfuron + iodosulfuron at the recommended label rate and from untreated control plants of the sensitive population, were analyzed. DNA was extracted using a customized kit (Chemagic Plant400 Kit, Perkin Elmer, Rodgau, Germany) and using a KingFisher™ Flex Magnetic Particle Processor (Thermo Fisher Scientific, Schwerte, Germany). Polymerase chain reaction (PCR) was performed on the genomic DNA (5 to 10 ng μL^−1^) using MangoTaq Polymerase (Bioline, Luckenwalde, Germany) and specific primer pairs (Table 3).

**Table 3:** Primer sets used to amplify partial *ALS* and *ACCase* genes to detect target‐site resistance (TSR) mutations in *P. annua*

Primer name	Primer sequence (5′‐ > 3′)	Product size (bp)	Targeted mutation site
Poa197‐for Poa197‐rev Poa197‐seq	CRATGGTCGCCATCACGG CTTGGTGATGGAACGGGTGAC GTGCCGATCATGCGG	98	ALS Pro‐197
Poa574‐for Poa574‐rev Poa574‐seq	CCTCCCYGTTAAGGTGATGATACT GTAWGTGTGCGCCCGATTG CCTTGTAAAACCTGTCCT	97	ALS Trp‐574
Poa1781‐for Poa1781‐rev Poa1781‐seq	CTCTTCTGTTATAGCGCACAAGAC CAACAGTTCGTCCAGTCACAA AGCAGCACTTCCATG	188	Inherited ACCase Ile‐1781
POAAN‐*ALS1*‐197‐for POAAN‐*ALS1*‐197‐rev	GCCACCGCGCTCCGCCCA CCTCCACGTCAAGGACCAGGTAA	436	*ALS1* Pro‐197
POAAN‐*ALS2*‐197‐for POAAN‐*ALS2*‐197‐rev	CCACCGCGCTCCGGCCGT TCGACGTCGAGGACCAGGTAGT	433	*ALS2* Pro‐197
POAAN‐*ALS1*‐574‐for POAAN‐*ALS1*‐574‐rev	TTCAGGAGTTGGCACTGATTCGTATTG GGCCCTGGAGTCTCAAGCATCT	268	*ALS1* Trp‐574
POAAN‐*ALS2*‐574‐for POAAN‐*ALS2*‐574‐rev	CAGGAGTTGGCACTGATTCGCATC GGCCCTGGAGTCTCAAGCATTG	266	*ALS2* Trp‐574

For pyrosequencing, primer sets were designed by retrieving the partial *ALS* and *ACCase* gene sequences of reference *P. annua* from the GenBank (KM388810.1 and MK992909.1, respectively) database. Gene fragments covering the two most common mutation sites, Pro‐197 and Trp‐574 of the *ALS* gene,^27^ and the inherited Ile‐1781 of the *ACCase* gene^24^ were amplified in a thermal cycler (T100 PCR thermal cycler, Bio‐Rad Laboratories, Feldkirchen, Germany). The PCR conditions used were initial denaturation for 3 min at 95 °C, followed by 40 cycles of denaturation at 95 °C for 10 s, annealing at 60 °C for 35 s and extension at 72 °C for 30 s and final extension at 72 °C for 5 min. The primer design was done to enable the same annealing temperature for all used primer sets. The success of the amplification step was checked using agarose gel electrophoresis. The PCR products were analyzed for single nucleotide polymorphisms (SNPs) using pyrosequencing on a PyroMark Q24 (Qiagen) using specific sequencing primers (Table 3). During the sequencing reaction, all incorporated nucleotides of a short region covering the position of interest were detected and reported by creating a pyrogram in a pyrorun file. Subsequently, the file was read by the PyroMark Q24 software (v. 2.0.8) and visually evaluated for mutations.

*P. annua* contains two *ALS* isoforms that are thought to be equally expressed and contribute to overall ALS activity.^15^ Each of these *ALS* homologs named *ALS1* and *ALS2* (GenBank accession numbers KT346395.1 and KT346396.1, respectively) can harbor resistance mutations. Thus, plants that appeared heterozygous mutated in pyrosequencing could have mutations in either or both *ALS* homologs, i.e., heterozygous mutant at both *ALS* isoforms, or homozygous mutant at one *ALS* isoform and homozygous wild‐type at the other. To locate resistance mutations in gene variants, the *ALS1* and *ALS2* reference sequences were aligned and nucleotide polymorphisms between both variants were: (a) used to create primers for isoform‐specific PCR (Table 3) and (b) used as diagnostic nucleotides to check the specificity of the PCR and to attribute the amplicons to the correct *ALS* gene variant. Only positions that showed a mutation in the pyrosequencing were analyzed (see section 3.2). Target DNA was amplified using the following PCR protocol: initial denaturation for 4 min at 95 °C, followed by 4 touch‐down cycles with denaturation at 95 °C for 15 s, annealing at 72 °C (−0.5 °C per cycle) for 30 s and extension at 72 °C for 1 min and 36 cycles of denaturation at 95 °C for 15 s, annealing at 70 °C for 30 s, and extension at 72 °C for 1 min, and final extension at 72 °C for 5 min. After the quality check on agarose gel, the amplicons and the corresponding forward (Pro‐197 fragment) or reverse (Trp‐574 fragment) primers were sent to a sequencing provider (Seqlab, Göttingen, Germany) for Sanger sequencing. The obtained nucleotide sequences were then analyzed using Geneious software (v. 9.1.8).

### 2.5 Efficacy of alternative non‐ALS herbicides

At the two‐to‐four leaf stage (BBCH 12–14), R and S populations were tested with the recommended label rate of four ACCase inhibitors pinoxaden (Axial Pro®, Syngenta, Cambridge, UK) at 30.3 g ai ha^−1^, fenoxaprop (Foxtrot EW® FMC Agro Ltd., Flintshire, UK) at 82.8 g ai ha^−1^, cycloxydim (Stratos® Ultra, BASF, Stockport, UK) at 150 g ai ha^−1^ and clethodim (Centurion® Max, Arysta LifeScience, Nogueres, France) at 120 g ai ha^−1^ and one EPSPS (5‐enolpyruvylshikimate‐3‐phosphate synthase) inhibitor glyphosate (Roundup® Flex, Bayer, Cambridge, UK) at 540 g ai ha^−1^. Each treatment had at least three replicates for each population, with at least six plants per replicate, and the entire experiment was repeated. Plant survival was assessed visually at 30 days post‐treatment. Surviving plants that continued to produce new shoots or tillers after treatment were recorded as resistant, and non‐recovering plants with symptoms of severe leaf chlorosis or no new active growth, and ultimately plant death were recorded as susceptible.

### 2.6 Statistical analysis

The statistical software R (v.3.6.3) was used to analyze the data. Dose–response models were fitted to the shoot fresh weight data using the *DRC* package and two models were chosen using the lack‐of‐fit *F‐*tests (*P* > 0.05).^28^ A four‐parameter Weibull‐1 model was used to model the data of mesosulfuron + iodosulfuron, and mesosulfuron + thiencarbazone, and a four‐parameter log‐logistic model was used to model the data of mesosulfuron + propoxycarbazone, pyroxsulam and propoxycarbazone. Model and residual non‐normality was adjusted using a Box‐Cox transformation when necessary. The fitted models estimated the growth rate GR_50_ value (i.e., the effective dose rate required to obtain a growth reduction of 50% relative to untreated plants) for the active ingredients in both co‐formulated and stand‐alone ALS inhibitors. The resistance index (RI) was calculated as the GR_50_ of the R population divided by the GR_50_ of the sensitive reference population.

## 3 RESULTS

### 3.1 Dose–response to ALS inhibitors

**Figure 1:**
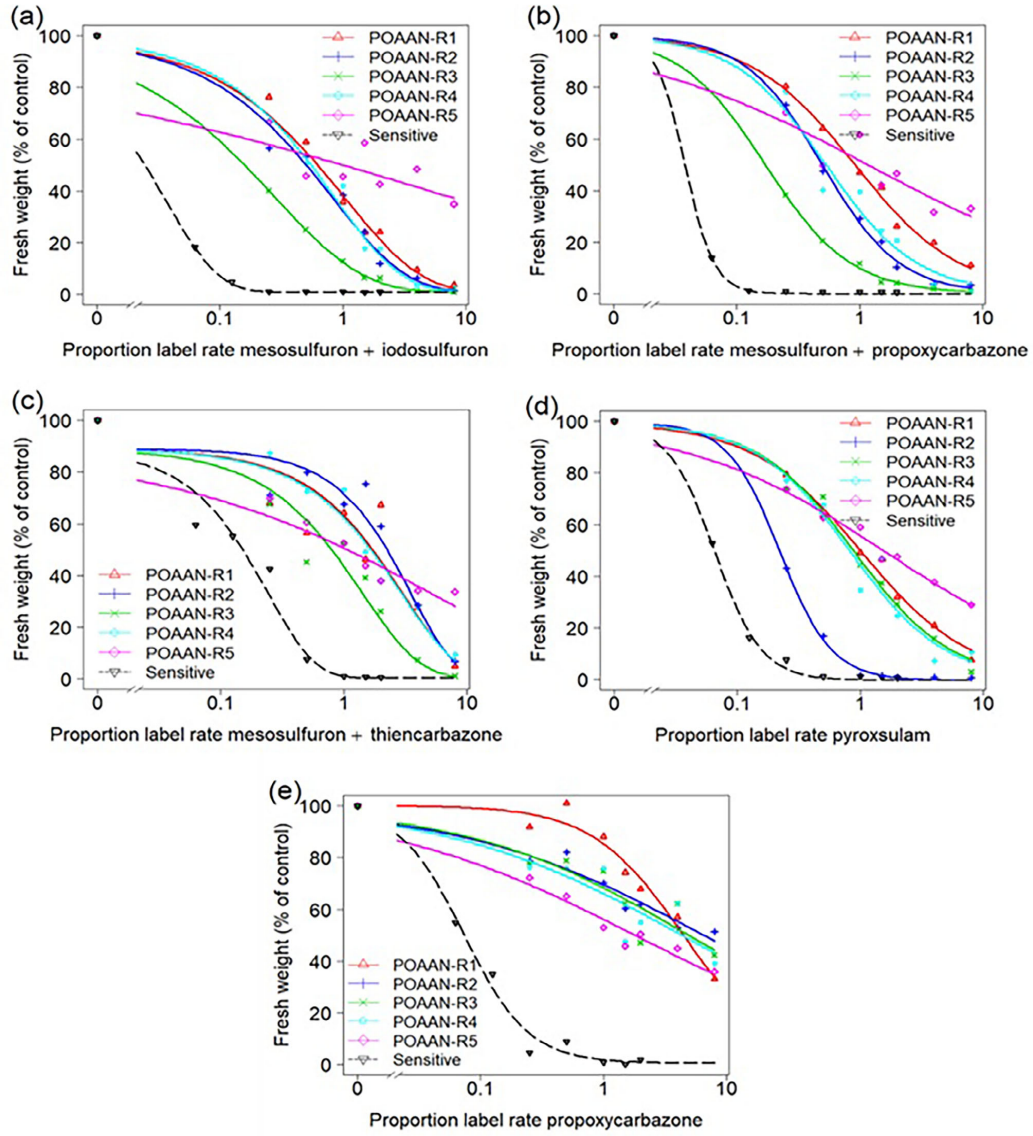
Dose–response curves of sensitive and resistant *P. annua* populations treated with a range of rates of ALS‐inhibiting herbicides. Treatments include: co‐formulated (a) SU (mesosulfuron + iodosulfuron), (b) SU + SCT (mesosulfuron + propoxycarbazone), (c) SU + SCT (mesosulfuron + thiencarbazone), and (d) TP (pyroxsulam), and stand‐alone (e) SCT (propoxycarbazone).

As expected, the S population was well controlled by all ALS inhibitors at rates equal to or below the recommended label rate, resulting in a biomass reduction of over 90% (Fig. 1). The GR_50_ values for the co‐formulated SU, SU + SCT and TP treatments were 0.4 + 0.1 g ai ha^−1^, 0.5 + 0.9 or 2.7 + 0.9 g ai ha^−1^ and 1.3 g ai ha^−1^, respectively (Table 4). For the stand‐alone SCT treatment, the GR_50_ value was 5.1 g ai ha^−1^.

**Table 4:** Estimated GR_50_ (standard errors) for each active ingredient (ai) in the co‐formulated (SU, SU + SCT and TP) and stand‐alone (SCT) ALS‐inhibiting herbicides for the sensitive and resistant *P. annua* populations. The resistance index (RI) was calculated as the ratio of GR_50_ values of R and S and only one RI value is presented

	Co‐formulated								Stand‐alone	
	Mesosulfuron + iodosulfuron		Mesosulfuron + propoxycarbazone		Mesosulfuron + thiencarbazone		Pyroxsulam		Propoxycarbazone	
Recommended label rate	**15 + 5 g ai ha** ^**−1**^		**14.9 + 22.3 g ai ha** ^**−1**^		**14.9 + 5 g ai ha** ^**−1**^		**18.8 g ai ha** ^**−1**^		**70 g ai ha** ^**−1**^	
Population	**GR**_**50**_ (**g ai ha** ^**−1**^)	**RI**	**GR**_**50**_ (**g ai ha** ^**−1**^)	**RI**	**GR**_**50**_ (**g ai ha** ^**−1**^)	**RI**	**GR**_**50**_ (**g ai ha** ^**−1**^)	**RI**	**GR**_**50**_ (**g ai ha** ^**−1**^)	**RI**
Sensitive	0.4 (0.10) + 0.1 (0.03)	‐	0.5 (0.47) + 0.9 (0.73)	‐	2.7 (0.58) + 0.9 (0.19)	‐	1.3 (0.11)	‐	5.1 (0.85)	‐
POAAN‐R1	9.2 (1.15) + 3.1 (0.38)	23.0	13.5 (1.29) + 20.3 (1.93)	27.0	30.7 (5.45) + 10.2 (1.78)	11.4	19.1 (2.04)	14.7	309.1 (51.31)	60.6
POAAN‐R2	7.4 (0.97) + 2.5 (0.32)	18.5	7.3 (0.62) + 11.0 (0.93)	14.6	38.6 (5.37) + 12.8 (1.77)	14.3	4.2 (0.43)	3.2	442.0 (198.37)	86.7
POAAN‐R3	2.4 (0.44) + 0.8 (0.15)	6.0	2.5 (0.46) + 3.8 (0.69)	5.0	14.3 (2.41) + 4.8 (0.79)	5.3	16.7 (1.67)	12.9	334.5 (124.41)	65.6
POAAN‐R4	8.1 (1.05) + 2.7 (0.35)	20.3	7.9 (0.77) + 11.8 (1.15)	15.8	29.7 (4.27) + 9.9 (1.48)	11.0	15.6 (1.53)	12.0	294.2 (111.27)	57.7
POAAN‐R5	14.1 (6.52) + 4.7 (2.17)	35.3	17.0 (3.20) + 25.5 (4.78)	34.0	26.9 (8.99) + 8.8 (2.96)	10.0	29.5 (4.96)	22.7	121.5 (43.22)	23.8

The R populations exhibited cross‐resistance between SU and SCT and between SU or SCT and TP (Fig. 1). With co‐formulated treatments (Fig. 1(a)–(d) and Table 4), POAAN‐R1, POAAN‐R4 and POAAN‐R5 showed high GR_50_ values and resistance indices (RI > 10) to SU, SU + SCT and TP, as confirmed by the lack of control, even with 8‐times the recommended label rate. POAAN‐R2 showed high resistance to SU and SU + SCT (RI >10) but low or evolving resistance (confirmed by a few surviving plants at the recommended label rate) to TP (RI = 3.2). Conversely, POAAN‐R3 showed high resistance to TP (RI >10), but moderate resistance (confirmed by the lack of control, with up to twice the recommended label rate) to SU and SU + SCT (RI >5).

The SCT‐only result showed all five POAAN‐R populations to have high resistance (RI >10) (Fig. 1(e) and Table 4). Despite the higher dose rates of active ingredient in the stand‐alone, it performs very poorly (Table 2).

### 3.2 Genetic analysis

Pyrosequencing analysis revealed heterozygous mutations at positions Pro‐197 and/or Trp‐574 in the *ALS* gene in all of the R populations, but not in the S population (Table 5).

**Table 5:** Amino acid (aa) substitutions identified at positions Pro‐197 and Trp‐574 of the *ALS* gene in *P. annua* populations. Eight plants per population were analyzed. The number of plants in which specific mutation(s) were detected are given in parenthesis. Sequence alignment analysis (*ALS*1 and *ALS*2) was conducted only on ALS positions that showed a mutation in the pyrosequencing. Mutant aa substitutions are in bold

	ALS pyrosequencing results				*ALS*1				*ALS*2			
	Pro (CCG)‐197		Trp (TGG)‐574		Pro (CCG)‐197		Trp (TGG)‐574		Pro (CCG)‐197		Trp (TGG)‐574	
Population	Codon	aa	Codon	aa	Codon	aa	Codon	aa	Codon	aa	Codon	aa
POAAN‐R1	C/A‐CG (8)	**Pro/Thr**	TGG (8)	Trp	CCG (8)	Pro			ACG (8)	**Thr**		
POAAN‐R2	C‐C/A‐G (8)	**Pro/Gln**	TGG (8)	Trp	CAG (8)	**Gln**			CCG (8)	Pro		
POAAN‐R3	C‐C/A‐G (5), C/A‐CG (3)	**Pro/Gln, Pro/Thr**	TGG (8)	Trp	CCG (3), CAG (5)	Pro, **Gln**			CCG (5), ACG (3)	Pro, **Thr**		
POAAN‐R4	C/A‐CG (7)	**Pro/Thr**	T‐G/T‐G (1)	**Trp/Leu**	CCG (8)	Pro	TGG (8)	Trp	ACG (7)	**Thr**	TGG (7), TTG (1)	Trp, **Leu**
POAAN‐R5	CCG (8)	Pro	T‐G/T‐G (8)	**Trp/Leu**			TGG (8)	Trp			TTG (8)	**Leu**
Sensitive	CCG (8)	Pro	TGG (8)	Trp								

Specific substitutions and mutant frequencies were identified in the POAAN‐R populations: Pro‐197‐Thr (100%) in POAAN‐R1, Pro‐197‐Gln (100%) in POAAN‐R2, a combination of Pro‐197‐Gln (62.5%) and Pro‐197‐Thr (37.5%) in POAAN‐R3, a combination of Pro‐197‐Thr (87.5%) and Trp‐574‐Leu (12.5%) in POAAN‐R4, and Trp‐574‐Leu (100%) in POAAN‐R5. It is important to note that, neither POAAN‐R3 nor POAAN‐R4 had combined mutations in the same plant (Table 5).

The sequence alignment analysis revealed that these mutations evolved independently in *ALS*1 or *ALS*2 homologs (Table 5). For POAAN‐R1, POAAN‐R3, POAAN‐R4 and POAAN‐R5, homozygous Pro‐197‐Thr or Trp‐574‐Leu substitutions occurred in the *ALS*2 isoform, while for POAAN‐R2 and POAAN‐R3, homozygous Pro‐197‐Gln substitutions occurred in the *ALS*1 isoform.

As expected, both R and S populations had a fixed Ile‐1781‐Leu substitutions (ATA to A/T‐TA) in the *ACCase* gene in all eight plants tested (data not shown).

### 3.3 Effect of alternative non‐ALS herbicides

All POAAN populations, which had a natural inherited ACCase mutation, were resistant (i.e., 100% of plants surviving) to pinoxaden, fenoxaprop and cycloxydim but sensitive (i.e., all treated plants died) to clethodim (Table 6), which is consistent with previous research.^2^, ^12^, ^25^, ^26^ In addition, all POAAN populations were also sensitive to glyphosate. Both the R and S populations of *P. annua* had similar responses (*P* > 0.05 for experiment *x* treatment interactions using ANOVA) to the recommended label rate of ACCase and EPSPS inhibitors.

**Table 6:** Effect of alternative non‐ALS herbicides at the recommended label rate on plant survival (%) of sensitive and resistant *P. annua* populations. Percent survival of each population was determined by dividing the total number of plants treated

	% plant survival				
	ACCase inhibitors				EPSPS inhibitors
Population	Pinoxaden	Fenoxaprop	Cycloxydim	Clethodim	Glyphosate
POAAN‐R1	100	100	100	0	0
POAAN‐R2	100	100	100	0	0
POAAN‐R3	100	100	100	0	0
POAAN‐R4	100	100	100	0	0
POAAN‐R5	100	100	100	0	0
Sensitive	100	100	100	0	0

## 4 DISCUSSION

This study has verified the genetic basis of herbicide resistance and quantified the cross‐resistance levels to ALS inhibitors, in five resistant *P. annua* populations. The populations originated from winter wheat fields that had a history of using SU, with or without TP, for grass weed control. Growers, who typically used 0.5‐ to 0.8‐times the recommended rate of these herbicides, had noticed ineffective weed control.

In resistant POAAN‐R populations, mutations in one of the two *ALS* isoforms explained the phenotypic resistance: Pro‐197‐Thr in *ALS*2 for POAAN‐R1, Pro‐197‐Gln in *ALS*1 for POAAN‐R2, Pro‐197‐Gln + Pro‐197‐Thr independently in *ALS*1 or *ALS*2 for POAAN‐R3, Pro‐197‐Thr + Trp‐574‐Leu independently in *ALS*2 for POAAN‐R4, and Trp‐574‐Leu in *ALS*2 for POAAN‐R5. This pattern suggests resistance resulted from independent selection within the individual population. TSR mechanisms are more prevalent in self‐pollinating polyploid weeds like *P. annua*;^29^, ^30^, ^31^, ^32^, ^33^, ^34^, ^35^, ^36^, ^37^, ^38^ as the accumulation and fixation of positive alleles increases with ploidy levels.^39^ Previous studies on ALS‐resistant *P*. annua, have identified Pro‐197‐Ser (conferring high SU resistance)^12^ or Ala‐205‐Phe (conferring high SU, SCT and TP resistance)^15^ in non‐arable settings, and Trp‐574‐Leu (conferring high SU, SCT and TP resistance)^2^, ^16^ in both non‐arable and arable settings. This study reports the first identification of Pro‐197‐Thr and/or Pro‐197‐Gln substitutions, as well as the stacked Pro‐197‐Thr + Trp‐574‐Leu substitutions, in *P. annua*.

In addition to the TSR mutation site, specific amino acid substitutions also influenced the ALS cross‐resistance spectrum, as indicated by the resistance indices recorded in the POAAN‐R populations. The Thr substitution in POAAN‐R1 and POAAN‐R4, or the Gln substitution in POAAN‐R2, both resulted in similarly high levels of SU and SU + SCT resistance (RI >10). However, these substitutions had different effects on TP resistance, with POAAN‐R1 and POAAN‐R4 exhibiting higher resistance levels (RI >10) compared to POAAN‐R2 (RI = 3.2) suggesting that the Thr substitution has a greater impact on TP resistance than the Gln substitution. This is further supported by the results in POAAN‐R3, which harbored both Thr and Gln substitutions. The Thr substitution, regardless of mutant frequency, gave high levels of TP resistance similar to POAAN‐R1 (RI >10), while Gln gave moderate levels of SU or SU + SCT resistance (RI >5 with low GR_50_ values), differing from POAAN‐R2 (RI >10). This suggests that, in *P. annua*, the frequency of Gln, or its combination with Thr, tends to affect SU and SU + SCT resistance less than Gln alone. As expected, the Trp‐574‐Leu substitutions in POAAN‐R5 was associated with high levels of cross‐resistance to SU, SU + SCT and TP (RI >10).

Thr or Gln substitutions at the Pro‐197 position have been widely reported in diploid species.^40^ Pro‐197‐Thr substitutions have been associated with SU and TP cross‐resistance in polyploid species such as *Echinochloa spp*. (barnyard grasses),^30^ and *Capsella bursa‐pastoris* (Shepherd's purse).^32^ In contrast, Pro‐197‐Gln has been associated with high SU resistance, but not TP resistance, in *Stellaria media* (common chickweed).^34^ The presence of different substitutions at the same ALS mutation site or the stacking of ALS TSR mutations are also relatively rare in polyploid species. However, substitutions such as Pro‐197‐Ser + Pro‐197‐Gln in *C. bursa‐pastoris* or multiple unexpected mutations such as Pro‐197‐Ser + Trp‐574‐Gly or Pro‐197‐Ser + Trp‐574‐Leu in *C. bursa‐pastoris*,^32^, ^33^ Pro‐197‐Ser + Trp‐574‐Leu or Pro‐197‐Thr + Trp‐574‐Leu in *S. media*,^35^, ^36^ and Ala‐122‐Asn + Trp‐574‐Leu in *E. crus‐galli* (L.) Beauv (barnyard grass)^29^ have been previously reported. The Trp‐574‐Leu substitution is known to confer high and broad resistance in both diploid and polyploid species.^40^

Among alternative non‐residual herbicides evaluated, both glyphosate and clethodim effectively controlled POAAN populations, though their practical use is more limited. Glyphosate is applied pre‐sowing or as a part of stale seedbed strategy,^1^ while clethodim is used within broad‐leaved crops (winter oilseed rape or beet) grown in rotations.^2^ While *P. annua* control in cereal crops has not been a challenge in this region to date, the increasing reliance on a single, resistance‐prone mode of action (ALS SU, SCT and TP inhibitors) for grass weed control will cause challenges. This situation differs from nearby regions (e.g., UK and mainland Europe) where *Alopecurus myosuroides* L. (black‐grass) or *Lolium multiflorum* L. (Italian ryegrass) presence results in growers using a robust pre‐emergence or autumn residual herbicide program (e.g., prosulfocarb, flufenacet, pendimethalin, *etc*.),^41^ which also addresses *P. annua*, but with less risk of resistance evolution. A return to the use of autumn‐applied residual herbicides, will provide *P. annua* control and eliminate the risk of selection of resistant *P. annua* biotypes by any subsequent use of ALS herbicides applied for *B. sterilis* or other grass weed control. However, this approach will be challenged in the future with the recent phase‐out of certain residual actives (e.g., metribuzin in 2024); flufenacet at risk of deregistration, and others, including chlortoluron, diflufenican and pendimethalin, listed as candidates for substitution in EU listings of registered herbicides. Cultural and non‐chemical control measures would also have limited effects due to *P. annua's* extended germination period, high seed production, and seed longevity, leaving the establishment of competitive crops and perhaps more inclusion of spring‐sown crops, as being the only other control measures possible.

## 5 CONCLUSION

This study contributes to the understanding of resistance patterns and underlying mechanisms to ALS‐inhibiting herbicides in *P. annua* populations from Ireland. The resistance profiles were associated with TSR mutations at Pro‐197 and/or Trp‐574, with specific substitutions (Thr and/or Gln in the Pro‐197 or Leu in the Trp‐574) in one of the two *ALS* isoforms conferring different levels of cross‐resistance to ALS inhibitors. Adoption of integrated weed management (IWM) approaches, including the use of residual herbicides, limiting the use of ALS chemistry and optimizing the use of limited cultural/non‐chemical practices alongside resistance monitoring, is essential to maintain the capacity to control *P. annua* into the future.

## CONFLICT OF INTEREST

No conflicts of interest have been declared.

## Data Availability

The data that support the findings of this study are available from the corresponding author upon reasonable request.
